# Precise-Spike-Driven Synaptic Plasticity: Learning Hetero-Association of Spatiotemporal Spike Patterns

**DOI:** 10.1371/journal.pone.0078318

**Published:** 2013-11-05

**Authors:** Qiang Yu, Huajin Tang, Kay Chen Tan, Haizhou Li

**Affiliations:** 1 Department of Electrical and Computer Engineering, National University of Singapore, Singapore, Singapore; 2 Institute for Infocomm Research, Agency for Science Technology and Research (A*STAR), Singapore, Singapore; 3 College of Computer Science, Sichuan University, Chengdu, China; 4 School of Electrical Engineering and Telecommunications, University of New South Wales, Sydney, Australia; SUNY Downstate MC, United States of America

## Abstract

A new learning rule (Precise-Spike-Driven (PSD) Synaptic Plasticity) is proposed for processing and memorizing spatiotemporal patterns. PSD is a supervised learning rule that is analytically derived from the traditional Widrow-Hoff rule and can be used to train neurons to associate an input spatiotemporal spike pattern with a desired spike train. Synaptic adaptation is driven by the error between the desired and the actual output spikes, with positive errors causing long-term potentiation and negative errors causing long-term depression. The amount of modification is proportional to an eligibility trace that is triggered by afferent spikes. The PSD rule is both computationally efficient and biologically plausible. The properties of this learning rule are investigated extensively through experimental simulations, including its learning performance, its generality to different neuron models, its robustness against noisy conditions, its memory capacity, and the effects of its learning parameters. Experimental results show that the PSD rule is capable of spatiotemporal pattern classification, and can even outperform a well studied benchmark algorithm with the proposed relative confidence criterion. The PSD rule is further validated on a practical example of an optical character recognition problem. The results again show that it can achieve a good recognition performance with a proper encoding. Finally, a detailed discussion is provided about the PSD rule and several related algorithms including tempotron, SPAN, Chronotron and ReSuMe.

## Introduction

With the same capability of processing spikes as biological neural systems, spiking neural networks (SNNs) [1]–[3] are more biologically realistic and computationally powerful than the traditional artificial neural networks (ANNs). Spikes are believed to be the principal feature in the information processing of neural systems, though the neural coding mechanism, i.e., how information is encoded in spikes still remains unclear. For example, many different neural codes have been introduced to describe how the spatiotemporal spikes convey the information of external stimuli, and among them rate code and temporal code [4] are the two most widely studied coding schemes. The rate code is a basic example of a neural code where information is conveyed through the spike count within a time window. Evidence to support the hypothesis of the rate code is demonstrated in [5], where a correlation of firing rates with sensory variables is shown. In the temporal code, the precise timing of each spike is considered. Recently, increasing experimental evidence suggests that neural systems use the exact time of spikes to convey information. For example, neurons are revealed to precisely respond to stimuli on a millisecond precision in the retina [6], [7], the lateral geniculate nucleus [8] and the visual cortex [9], [10]. These observations support the hypothesis of the temporal code. Additionally, recent studies also show that the temporal coding scheme can offer significant computational advantages over the rate coding scheme [11]–[13]. However, the complexity of processing temporal codes [14], [15] might limit their usage in SNNs, which demands the development of efficient learning algorithms.

Supervised learning was proposed as a successful concept of information processing [16]. Neurons are driven to respond at desired states under a supervisory signal, and an increasing body of evidence shows that this kind of learning is exploited by the brain [17]–[20]. Supervised mechanism has been widely used to develop various learning algorithms for processing spatiotemporal spike patterns in SNNs [15], [21]–[27].

Some of the existing supervised learning rules, such as spike-driven synaptic plasticity [21], are formulated in a rate-based framework and are not feasible for the processing of precise-timing spike patterns. In the spike-driven synaptic plasticity approach, the learning process is supervised and stochastic, meaning that a teacher signal steers the output neuron to a desired firing rate. According to this algorithm, synaptic weights are modified upon the arrival of presynaptic spikes, considering the state of the postsynaptic neuron's potential and its recent firing activity.

SpikeProb [22] is one of the first supervised learning algorithms for processing precise spatiotemporal patterns in SNNs. It is a gradient descent based learning rule, which can solve nonlinear classification tasks by emitting single spikes at the desired firing time. However, in its original form, SpikeProb cannot learn to reproduce a multi-spike train. The tempotron rule [15], another gradient descent approach that is evaluated to be efficient for binary temporal classification tasks, cannot output multiple spikes either. As the tempotron is designed mainly for pattern recognition, it is unable to produce precise spikes. The time of the tempotron's output spike seems to be arbitrary and does not carry information. By this nature, the output of a tempotron cannot serve as the input for another tempotron. To produce a desired spike train, several learning algorithms have been proposed such as ReSuMe [23], [28], Chronotron [24] and SPAN [25]. These three learning rules are all capable of training a neuron to generate a desired spike train in response to an input stimulus. The ReSuMe rule is based on a learning window concept similar to spike-timing-dependent plasticity (STDP) [29], [30]. The ReSuMe interprets the Widrow-Hoff (WH) rule [16] through interaction of two biological processes: Hebbian and anti-Hebbian learning. In the Chronotron, two learning rules are introduced. One is analytically-derived (E-learning) and another one is heuristically-defined (I-learning). The I-learning rule is more biologically plausible but comes with less memory capacity than the E-learning rule. The performance of the I-learning rule depends on the weight initialization, where initial zero values can cause information loss from the corresponding afferent neurons. The E-learning rule and the SPAN rule are both based on an error function of the difference between the actual output spike train and the desired spike train. Their applicability is therefore limited to the tractable error evaluation, which might be unavailable in actual biological networks and inefficient from a computational point of view. These arithmetic-based rules can reveal explicitly how SNNs can be trained but the biological plausibility of the error calculation is somewhat questionable.

In this paper, we propose an alternative learning mechanism called Precise-Spike-Driven (PSD) synaptic plasticity, that is able to learn the association between precise spike patterns. Similar to ReSuMe [23] and SPAN [25], the PSD rule is derived from the WH rule but based on a different interpretation. The PSD rule is derived analytically based on converting the spike trains into analog signals by applying the spike convolution method. Such an approach is rarely reported in the existing learning rule studies [25]. Synaptic adaptation in the PSD is driven by the error between the desired and the actual output spikes, with positive errors causing long-term potentiation (LTP) and negative errors causing long-term depression (LTD). The amount of adaptation depends on an eligibility trace determined by the afferent spikes. Without complex error calculation, the PSD rule provides an efficient way for processing spatiotemporal patterns. We show that the PSD rule inherits the advantageous properties of both arithmetic-based and biologically realistic rules, being simple and efficient for computation, and yet biologically plausible. Furthermore, the PSD is an independent plasticity rule that can be applied to different neuron models. This straightforward interpretation of the WH rule also provides a possible direction for further exploitation of the rich theory of ANNs, and minimizes the gap between the learning algorithms of SNNs and the traditional ANNs.

Various properties of the PSD rule are investigated through an extensive experimental analysis. In the first experiment, the basic concepts of the PSD rule are demonstrated, and its learning ability on hetero-association of spatiotemporal spike pattern is investigated. In the second experiment, the PSD rule is shown to be applicable to different neuron models. Thereafter, experiments are conducted to analyze the learning rule regarding its robustness against noisy conditions, its memory capacity, effects of the learning parameters and its classification performance. The capability of the PSD rule is further demonstrated on a practical example of an optical character recognition (OCR) problem. Finally, a detailed discussion about the PSD rule and several related algorithms including tempotron, SPAN, Chronotron and ReSuMe is presented.

## Methods

In this section, we begin by presenting the spiking neuron models. We then describe the PSD rule for learning hetero-association between the input spatiotemporal spike patterns and the desired spike trains.

### Spiking Neuron Model

As the third generation neuron model, spiking neurons raise the level of biological realism by utilizing spikes [3]. The spiking neurons perform computation using the precise timing spikes, and offer improvements over the traditional neural models in terms of accuracy and computational power [31]. There are several kinds of spiking neuron models such as the integrate-and-fire (IF) model [1], the resonate-and-fire model [32], the Hodgkin-Huxley model [33], and the Izhikevich (IM) model [34]. Because the IF model is simple and computationally effective, it has become the most widely used spiking neuron model [15], [21], [22], [28], [35]–[37], despite other more biologically realistic models.

For the sake of simplicity, the leaky integrate-and-fire (LIF) model is firstly considered. The dynamics of each neuron evolves according to the following equation:
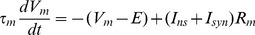
(1)where 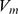 is the membrane potential, 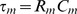 is the membrane time constant, 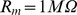 and 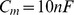 are the membrane resistance and capacitance, respectively, 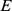 is the resting potential, 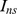 and 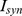 are the background current noise and synaptic current, respectively. When 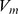 exceeds a constant threshold 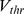, the neuron is said to fire, and 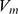 is reset to 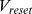 for a refractory period 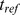. We set 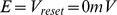 and 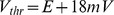 for clarity, but any other values as 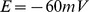 will result in equivalent dynamics as long as the relationships among 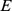, 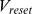 and 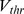 are kept.

For the postsynaptic neuron, we model the input synaptic current as:
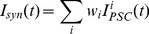
(2)where 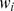 is the synaptic efficacy of the 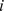-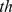 afferent neuron, and 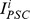 is the un-weighted postsynaptic current from the corresponding afferent.
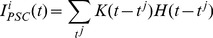
(3)where 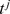 is the time of the 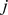-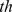 spike emitted from the 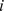-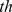 afferent neuron, 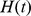 refers to the Heaviside function, 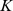 denotes a normalized kernel and we choose it as:
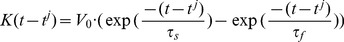
(4)where 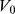 is a normalization factor such that the maximum value of the kernel is 1, 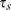 and 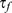 are the slow and fast decay constants respectively, and their ratio is fixed at 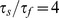.

**Figure 1:**
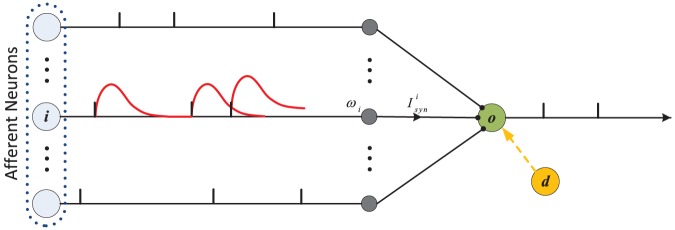
Illustration of the neuron structure. The afferent neurons are connected to the postsynaptic neuron through synapses. Each emitted spike from afferent neurons will trigger a postsynaptic current (PSC). The membrane potential of the postsynaptic neuron is a weighted sum of all incoming PSCs from all afferent neurons. The yellow neuron denotes the instructor which is used for learning.

Fig. 1 illustrates the neuron structure. Each spike from the afferent neuron will result in a postsynaptic current (PSC). The membrane potential of the postsynaptic neuron is a weighted sum of all incoming PSCs over all afferent neurons.

In addition to the LIF model, we also investigate the flexibility of the PSD rule to different neuron models. For this, we use the IM model [34], where the dynamics of the IM model is described as:
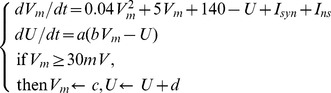
(5)where 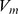 again represents the membrane potential. 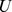 is the membrane recovery variable. The synaptic current (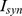) is in the same form as described before, and 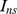 again represents the background noise. The parameters 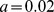, 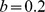, 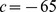 and 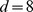 are chosen such that the neuron exhibits a regular spiking behavior which is the most typical behavior observed in cortex [34].

For computational efficiency, the LIF model is used in the following studies, unless otherwise stated.

### PSD Learning Rule

In this section we describe in detail the PSD learning rule. Note that the spiking neuron models were developed from the traditional neuron models. In a similar way, we develop the learning rule for spiking neurons from traditional algorithms. Inspired by [25], we derive the proposed rule from the common Widrow-Hoff (WH) rule. The WH rule is described as:
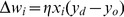
(6)where 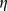 is a positive constant referring to the learning rate, 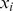, 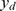 and 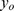 refer to the input, the desired output and the actual output, respectively.

Note that because the WH rule was introduced for the traditional neuron models such as perceptron, the variables in the WH rule are regarded as real-valued vectors. In the case of spiking neurons, the input and output signals are described by the timing of spikes. Therefore, a direct implementation of the WH rule does not work for spiking neurons. This motivates the development of the PSD rule.

A spike train is defined as a sequence of impulses triggered by a particular neuron at its firing time. A spike train is expressed in the form of:
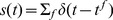
(7)where 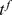 is the 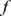-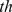 firing time, and 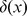 is the Dirac function: 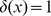 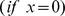 or 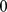 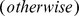. Thus, the input, the desired output and the actual output of the spiking neuron are described as:
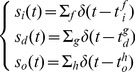
(8)


The products of Dirac functions are mathematically problematic. To solve this difficulty, we apply an approach called spike convolution. Unlike the method used in [25], which needs a complex error evaluation and requires spike convolution on all the spike trains of the input, the desired output and the actual output, we only convolve the input spike trains.
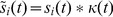
(9)where 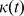 is the convolving kernel, which we choose to be the same as Eq. (4). In this case, the convolved signal is in the same form as 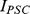 in Eq. (3). Thus, we use 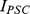 as the eligibility trace for the weight adaptation. The learning rule becomes:
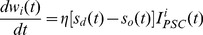
(10)


**Figure 2:**
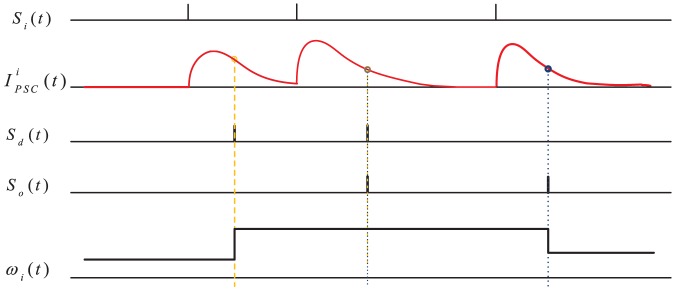
Illustration of the weight adaptation. 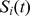
 is the presynaptic spike train. 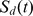 and 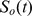 are the desired and the actual postsynaptic spike train, respectively. 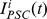 is the postsynaptic current and can be referred to as the eligibility trace for the adaptation of 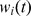. A positive error, where the neuron does not spike at the desired time, causes synaptic potentiation. A negative error, where the neuron spikes when it is not supposed to, results in synaptic depression. The amount of adaptation is proportional to the postsynaptic current. There will be no modification when the actual output spike fires exactly at the desired time. This figure is inspired from [28].

Eq. (10) formulates an online learning rule. The dynamics of this learning rule is illustrated in Fig. 2. It can be seen that the polarity of the synaptic changes depends on three cases: (1) a positive error (corresponding to a miss of the spike) where the neuron does not spike at the desired time, (2) a zero error (corresponding to a hit) where the neuron spikes at the desired time, and (3) a negative error (corresponding to a false-alarm) where the neuron spikes when it is not supposed to.

Thus, the weight adaptation is triggered by the error between the desired and the actual output spikes, with positive errors causing long-term potentiation and negative errors causing long-term depression. No synaptic change will occur if the actual output spike fires at the desired time. The amount of synaptic changes is determined by the current 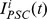.

With the PSD learning rule, each of the variables involved has its own physical meaning. Moreover, the weight adaptation only depends on the current states. This is different from rules involving STDP, where both the pre- and post-synaptic spiking times are stored and used for adaptation.

By integrating Eq. (10), we get:
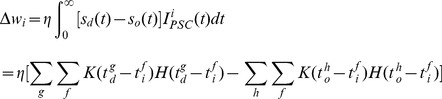
(11)


This equation could be used for trial learning where the weight modification is performed at the end of the pattern presentation.

In order to measure the distance between two spike trains, we use the van Rossum metric [38] but with a different filter function as described in Eq. (4). This filter is used to compensate for the discontinuity of the original filter function. The distance can be written as:
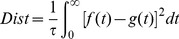
(12)where 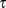 is a free parameter (we set 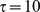 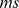 here), 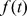 and 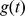 are filtered signals of the two spike trains that are considered for distance measurement. More details can be found in [38].

Noteworthily, this distance parameter 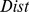 is not involved in the PSD learning rule, but is used for measuring and analyzing the performance of the learning rule, which reflects the dissimilarity between the desired and the actual spike trains. In the following experiments, different values of 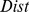 are used for analysis depending on the problems. For single-spike and multi-spike target trains, we set 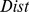 to be 0.2 and 0.5, respectively, corresponding to an average time difference of around 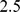 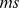 for each pair of the actual and desired spikes. Smaller 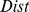 can be used if exact association is the main focus, e.g., 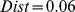 corresponds to a time difference about 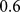 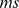, where no obvious dissimilarity can be seen between the two spike trains.

## Results

In this section, several experiments are presented to demonstrate the characteristics of the PSD rule. The basic concepts of the PSD rule are first examined, by demonstrating its ability to associate a spatiotemporal spike pattern with a target spike train. Furthermore, we show that the PSD has desirable properties, such as generality to different neuron models, robustness against noise and learning capacity. The effects of the parameters on the learning are also investigated. Then, the application of the proposed algorithm to the classification of spike patterns is also shown, with the final experiment demonstrating its performance on a practical OCR task.

### Association of Single-Spike and Multi-Spike Patterns

This experiment is devised to demonstrate the ability of the proposed PSD rule for learning a spatiotemporal spike pattern. The neuron is trained to reproduce spikes that fire at the same spiking time of a target train.

#### Experiment setup

The neuron is connected with 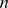 afferent neurons, and each fires a single spike within the time interval of 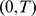. Each spike is randomly generated with a uniform distribution. We set 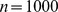, 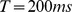 here. To avoid a single synapse dominating the firing of the neuron, we limit the weight below 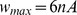. The initial synaptic weights are drawn randomly from a normal distribution with mean value of 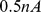 and a standard deviation of 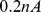. For the learning parameters, we set 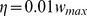 and 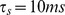. The target spike train can be randomly generated, but for simplicity, we specify it as 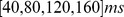. In this way, the spikes are evenly distributed over the whole interval 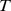.

#### Learning process

**Figure 3:**
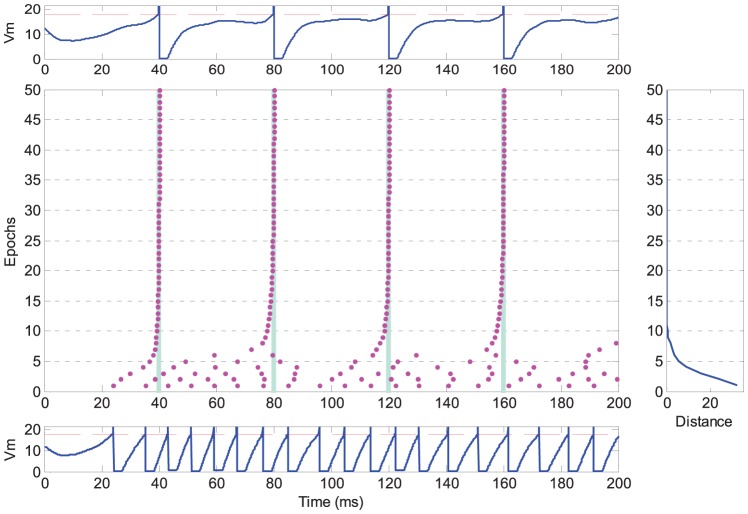
Illustration of the temporal sequence learning of a typical run. The neuron is connected with 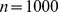 synapses, and is trained to reproduce spikes at the target time (denoted as light blue bars in the middle). The bottom and top show the dynamics of the neuron's potential before and after learning, respectively. The dashed red lines denote the firing threshold. In the middle, each spike is denoted as a dot. The right figure shows the distance between the actual output spike train and the target spike train.

Fig. 3 illustrates a typical run of the learning. Initially, the neuron is observed to fire at any arbitrary time and with a firing rate different from the target train, resulting in a large distance value. The actual output spike train is quite different from the target train at the beginning. During the learning process, the neuron gradually learns to produce spikes at the target time, and that is also reflected by the decreasing distance. After finishing the first 10 epochs of learning, both the firing rate and the firing time of the output spikes match those in the target spike train. The dynamics of neuron's membrane potential is also shown in Fig. 3. Whenever the membrane potential exceeds the threshold, a spike is emitted and the potential is kept at reset level for a refractory period. The detailed mathematical description governing this behaviour was presented previously in the section on the Spiking Neuron Model.

This experiment shows the feasibility of the PSD rule to train the neuron to reproduce a desired spike train. After several learning epochs, the neuron can successfully spike at the target time. In other words, the proposed rule is able to train the neuron to associate the input spatiotemporal pattern with a desired output spike train within several training epochs. The information of the input pattern is stored by a specified spike train.

#### Causal weight distribution

**Figure 4:**
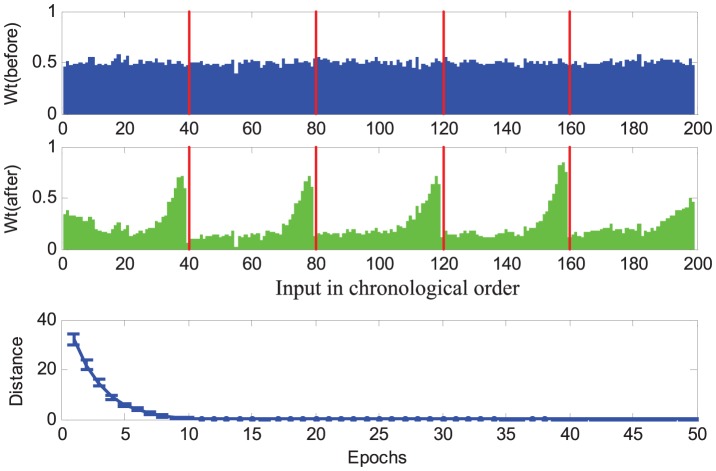
Effect of the learning on synaptic weights and the evolution of distance along the learning process. The top and the middle show the averaged weights before and after learning, respectively. The height of each bar in the figure reflects the corresponding synaptic strength. All the afferent neurons are chronologically sorted according to their spike time. The target spikes are overlayed on the weights figure according to their time, and are denoted as red lines. The bottom shows the averaged distance between the actual spike train and the desired spike train along the learning process. All the data are averaged over 100 runs.

We further examine how the PSD rule drives the synaptic weights and the evolution of the distance between the actual and the target spike trains. In order to guarantee statistical significance, the task described in Fig. 3 is repeated 100 times. Each time is referred to as one run. At the initial point of each run, different random weights are used for training. As can be seen from Fig. 4, the initial weights are normally distributed around 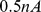, which reflects the fact that there are no significant differences among the input synapses. This initial distribution of weights is expected due to the experimental setup. After learning, a causal connectivity is established. According to the learning rule, the synapses that fire temporally close to the time of the target spikes are potentiated. Those synapses that result in undesired output spikes are depressed. This temporal causality is clearly reflected on the distribution of weights after learning (Fig. 4). Among those causal synapses, the one with a closer spiking time to the desired time normally has a relatively higher synaptic strength. The synapses firing far from the desired time will have lower causal effects. Additionally, the evolution of distance along the learning shows that the PSD rule successfully trains the neuron to reproduce the desired spikes in around ten epochs. The results also validate the efficiency of the PSD learning rule in accomplishing the single association task.

#### Adaptive learning performance

**Figure 5:**
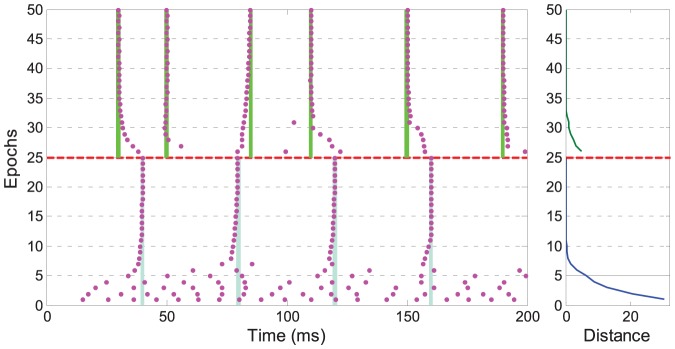
Illustration of the adaptive learning of the changed target trains. Each dot denotes a spike. At the beginning, the neuron is trained to learn one target (denoted by the light blue bars). After 25 epochs of learning (the dashed red line), the target is changed to another randomly generated train (denoted by the green bars). The right figure shows the distance between the actual output spike train and the target spike train along the learning process.

At the beginning, the neuron is trained to learn a target train as in the previous tasks. After one successful learning, the target spike train is changed to another arbitrarily generated train, where the precise spike time and the firing rate are different from the previous target. We discover that, with the PSD learning rule, we successfully train the neuron to learn the new target within several epochs. As shown in Fig. 5, during learning, the neuron gradually adapts its firing status from the old target to the new target.

#### Learning multiple spikes

**Figure 6:**
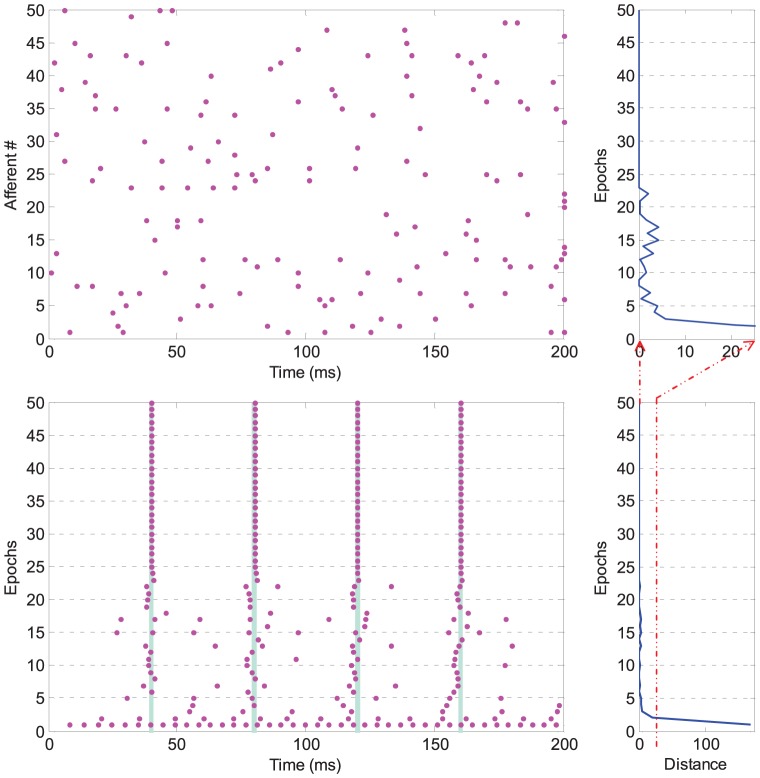
Illustration of a typical run for learning multi-spike pattern. Each dot denotes a spike. The top left shows the input spikes from the first 50 afferent neurons out of 1000. Each synaptic input is generated by a homogeneous Poisson process with a random rate from 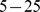 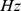. The bottom left shows the neuron's output spikes. The right column shows the distance between the actual output spike train and the target spike train along learning.

In the scenario considered above, all afferent neurons are supposed to fire only once during the entire time window. The applicability of the PSD rule is not limited to this single spike code. We further illustrate the case where each synaptic input transmits multiple spikes during the time window. We again use the same setup as above, but each synaptic input is now generated by a homogeneous Poisson process with a random rate ranging from 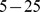 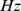. Multiple spikes increase the difficulty of the learning since these spikes interfere with the local learning processes [28]. As shown in Fig. 6, the learning although slower, is again successful. The interference of local learning processes results in fluctuations of the output spikes around the target time. In the subsequent learning epochs, the neuron gradually converges to spiking at the target time. This experiment demonstrates that the PSD rule deals with multiple spikes quite well. Compared to multiple spikes, the single spike code is simple for analysis and efficient for computation. Thus, for simplicity, we use the single spike code in the following experiments where each afferent neuron fires only once during the time window.

These experiments clearly demonstrate that the PSD rule is capable of training the neuron to fire at the desired time. The causal connectivity is established after learning with this rule. In the following sections, some more challenging learning scenarios are taken into consideration to further investigate the properties of the PSD rule.

### Generality to Different Neuron Models

**Figure 7:**
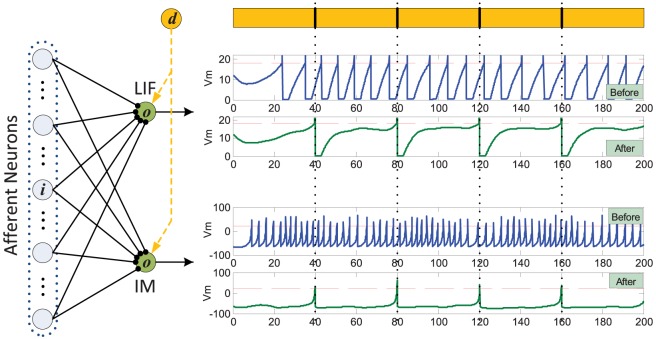
Learning with different spiking neuron models. The LIF and IM neuron models are considered. The left panel shows the connection setup of the experiment. Both the two neurons are connected to the same 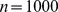 afferent neurons, and are trained to reproduce target spikes (denoted by the yellow parts). The right panel shows the dynamics of neurons' potential before and after learning. The dashed red lines denote the firing threshold.

We carry out this experiment to demonstrate that the PSD learning rule is independent of the neuron model. In this experiment, we only compare the results of learning association for the LIF and IM neuron models that were described previously. For a fair comparison, both neurons are connected to the same afferent neurons, and they are trained to reproduce the same target spike train. The setup for generating the input spatiotemporal patterns is the same as the experiment in Fig. 5. The connection setup is illustrated in Fig. 7. Except for the neuron dynamics described in Eq. (1) and Eq. (5) respectively, all the other parameters are the same for the two neurons.

The dynamic difference between the two types of spiking neuron models is clearly demonstrated in Fig. 7. Although the neuron models are different, both of the neurons can be trained to successfully reproduce the target spike train with the proposed PSD learning rule. It is seen that the two neurons fire at arbitrary time before learning, while after learning they fire spikes at the desired time.

In the PSD rule, synaptic adaptation is triggered by both the desired spikes and the actual output spikes. The amount of updating depends on the presynaptic spikes firing before the triggering spikes. That is to say, the weight adaptation of our rule is based on the correlation between the spiking time only. This suggests the PSD has the generality to work with various neuron models, a capability similar to that of the ReSuMe rule [28].

### Robustness to Noise

In previous experiments, we only consider the simple case where the neuron is trained to learn a single pattern under noise-free condition. However, the reliability of the neuron response could be significantly affected by noise. In this experiment, two noisy cases are considered: stimuli noise and background noise.

#### Experiment setup

In this experiment, a single LIF neuron with 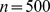 afferent neurons is tested. Initially, a set of 10 spike patterns are randomly generated as in previous experiments. These 10 spike patterns are fixed as the templates. The neuron is trained for 400 epochs to associate all patterns in the training set with a desired spike train (the same train as is used before). Two training scenarios are considered in this experiment, i.e., deterministic training (in the noise-free condition) and noisy training. In the testing phase, a total number of 200 noise patterns are used. Each template is used to construct 20 testing patterns. We determine the association to be correct, if the distance between the output spike train and the desired spike train is lower than a specified level (0.5 is used here).

#### Input jittering noise

**Figure 8:**
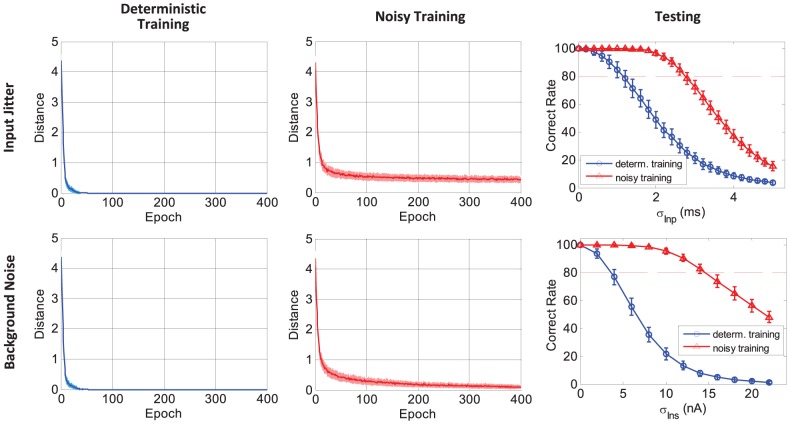
Robustness of the learning rule against jittering noise of input stimuli and background noise. The top row presents the case where the noise comes from the input spike jitters. The bottom row presents the case of background noise. The neuron is trained under noise-free conditions (denoted as deterministic training), or is trained under noisy conditions (denoted as noisy training). In the training phase (left two columns), the neuron is trained for 400 epochs. Along the training process, the average distance between the actual output spike train and the desired spike train is shown. The standard deviation is denoted by the shaded area. In the testing phase (right column), the generalization accuracies of the trained neuron on different levels of noise patterns are presented. Both the average value and the standard deviation are shown. All the data are averaged over 100 runs.

In the case of input jittering noise, a Gaussian jitter with a standard deviation (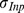) is added to each input spike to generate the noise patterns. The strength of the jitter is controlled by the standard deviation of the Gaussian. The top row in Fig. 8 shows the learning performance. In the deterministic training, the neuron is trained purely with the initial templates. In the noisy training, a noise level of 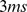 is used. Different levels of noise are used in the testing phase to evaluate the generalization ability. For the deterministic training, the output stabilizes quickly and can exactly converge to the desired spike train within tens of learning epochs. However, the generalization accuracy decreases quickly with the increasing jitter strength. In the scenario of noisy training, although the training error cannot become zero, a better generalization ability is obtained. The neuron can successfully reproduce the desired spike train with a relatively high accuracy when the noise strength is not higher than the one used in the training. In conclusion, the neuron is less sensitive to the noise if the noisy training is performed.

#### Background current noise

In this case, the background current noise (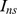) is considered as the noise source. The mean value of 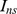 is assumed zero, and the strength of the noise is determined by its variance (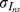). A strength of 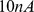 noise is used in the noisy training. We report the results in the bottom row of Fig. 8. Similar results are obtained as with the first case. Although the output can quickly converge to zero error in the deterministic training, the generalization performance is quite sensitive to the noise. The association accuracy drops quickly when the noise strength increases. When the neuron is trained with noise patterns, it becomes less sensitive to the noise. A relatively high accuracy can be obtained with a noise level up to 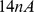.

This experiment shows that the trained neuron under noise-free conditions will be significantly affected by noise. Such an influence of noise on the timing accuracy and reliability of the neuron response has been considered in many studies [15], [24], [25], [27], [28], [39]. Under the noisy training, the trained neuron demonstrates high robustness against the noise. The noisy training enables the neuron to reproduce desired spikes more reliably and precisely.

### Learning Capacity

As used for the perceptron [40] and tempotron [15], [26] learning rules, the ratio of the number of random patterns (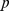) that a neuron can correctly classify over the number of its synapses (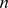), 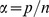, is used to measure the memory load. An important characteristic of a neuron's capacity is the maximum load that it can learn. In this experiment, the memory capacity of the PSD rule is investigated.

#### Experiment setup

We devise an experiment that has a similar setup to that in [25]. A number of 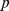 patterns are randomly generated in the same process as previous experiments, where each pattern contains 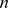 spike trains and each train has a single spike. The patterns are randomly and evenly assigned to 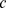 different categories. Here we choose 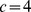 for this experiment. A single LIF neuron is trained to memorize all patterns correctly in a maximum number of 500 training epochs. The neuron is trained to emit a single spike at a specified time for patterns from each category. The desired spikes for the 4 generated categories are set to the time of 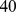, 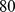, 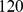 and 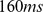, respectively. A pattern is considered to have been correctly memorized by the neuron if the distance between the actual spike train and the desired train is below 0.2. The learning process is considered a failure if the number of training epochs reaches the maximum number.

#### Maximum load factor

**Figure 9:**
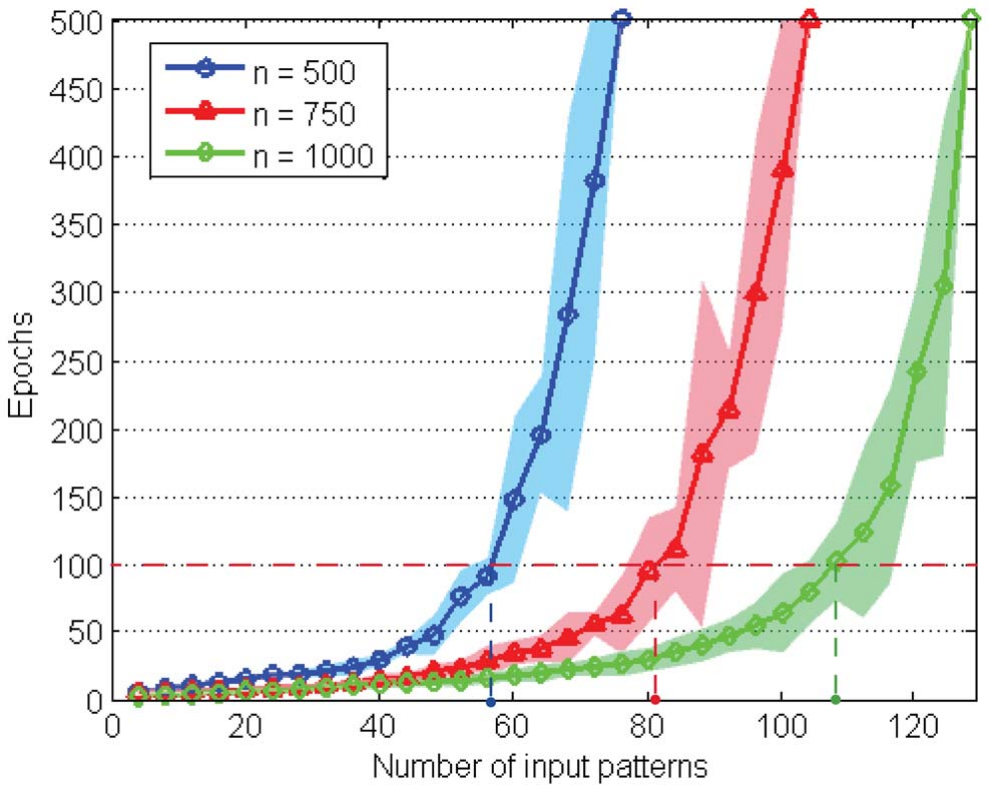
The memory capacity of the PSD rule with different numbers of synapses. The neuron is trained to memorize all patterns correctly in a maximum number of 500 epochs. The reaching points of 500 epochs are regarded as failure of the learning. The cases of 500, 750 and 1000 synapses are denoted by blue, red and green parts, respectively. The marked lines denote average learning epochs and the shaded areas show the standard deviation. The dashed line at 100 epochs is used for evaluating the efficient load 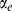 described in the main text. All the data are averaged over 100 runs.

Fig. 9 shows the results of the experiment for the case of 500, 750 and 1000 afferent neurons, respectively. All the data are averaged over 100 runs. In each run, different initial weights are used. As seen from Fig. 9, the number of epochs required for the training increases slightly as the number of patterns increases when the load is not too high, but a sharp increase of learning epochs occurs after a certain high load. This suggests that the task becomes tougher with an increasing load. It is also noted that a larger number of synapses leads to a bigger memory capacity for the same neuron. It is reported that the maximum load factors for 500, 750 and 1000 synapses are 0.144, 0.133 and 0.124, respectively.

#### Efficient load factor

Besides the maximum load factor, we heuristically define another factor, the efficient load 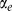. As described above, the neuron can perform the task efficiently with a relatively high load when the number of patterns does not exceed a certain value (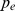). The efficient load is denoted as 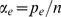. When the load is below 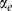, the neuron can reliably memorize all patterns with a small number of training epochs. There are different ways to define 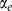. We show two possible ways. One is to derive the definition from a mathematical calculation such as 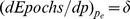, where 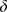 is a specified value (for example 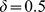). A simpler method is where a specified number of training epochs is used. The corresponding number of patterns that can be correctly learnt is considered as 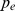. For simplicity, we use the latter as an example for demonstration and the specified number of epochs is set to 100. As seen from Fig. 9, the efficient load factors for 500, 750 and 1000 synapses are 0.112, 0.109 and 0.108, respectively. Surprisingly, these efficient load factors seem to all be around a stable value which only changes slightly across different numbers of synapses. This fixed value of efficient load factor for different values of 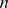 indicates that the number of patterns that a neuron can efficiently memorize grows linearly with the number of afferent synapses. It is worth noting that the concept of efficient load factor 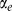 provides an important guideline for choosing the load of patterns when a reliable and efficient training is required.

### Effects of Learning Parameters

Two of the major parameters involved in the PSD learning rule are the learning rate 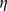 and the decay constant 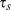. In this section, we aim to investigate the effects of these parameters on the learning process.

#### Small 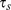 results in strong causal weight distribution

**Figure 10:**
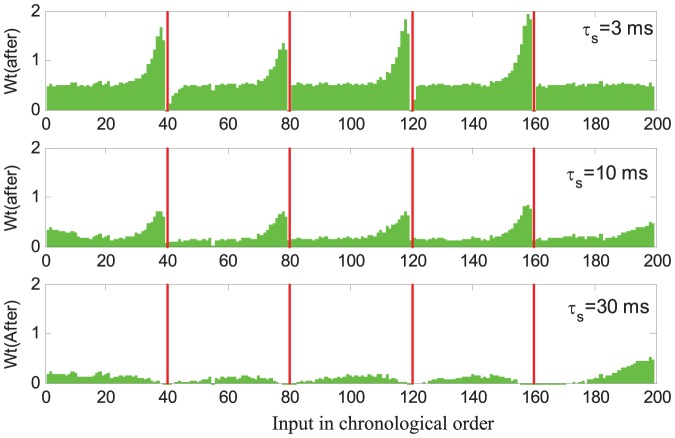
Effect of decay constant 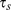 on the distribution of weights. The averaged weights after learning are shown. The height of each bar reflects the synaptic strength. The afferent neurons are chronologically sorted according to their spike time. The target spikes are overlayed and denoted as red lines. Cases of 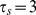, 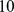 and 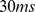 are depicted. All the data are averaged over 100 runs.

As a decay constant, 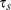 is an important parameter involved in the postsynaptic current. It determines how long a presynaptic spike will still have causal effect on the postsynaptic neuron. In the phase of synaptic adaptation, 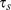 also determines the magnitude of modification on the synaptic weights at the time of a triggering spike. Thus, 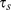 will affect the distribution of weights after the training. To look into this effect, we conduct an experiment with a similar setup as in Fig. 4 but with different values of 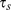. Here we choose 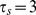, 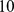 and 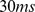. As can be seen from Fig. 10, a smaller 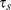 (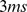) can result in a very uneven distribution with only a few synapses being given relatively higher weights. A flat distribution is obtained with an increasing 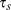. This is because 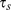 determines how long the causal effect of an afferent spike will sustain. A smaller 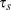 means that only the nearer neighbors are involved in generating the desired spikes, hence resulting in a smaller number of causal synapses. With a smaller number of causal synapses, a higher synaptic strength will be required to generate spikes at the desired time. On the other hand, with a larger 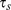, a wider range of causal neighbors can contribute to generating the desired spikes, and therefore a lower synaptic strength will be sufficient. The synaptic strength and distribution for different values of 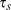 are obtained as in Fig. 10.

#### Effects of both 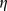 and 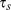 on the learning

**Figure 11:**
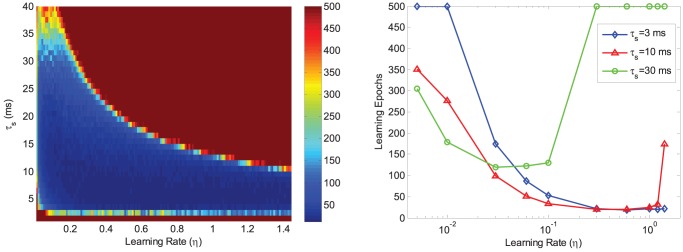
Effects of 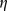 and 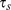 on the learning. The neuron is trained in a maximum number of 500 epochs to correctly memorize a set of 10 spike patterns. The average learning epochs are recorded for each pair of 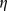 and 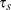. The reaching points of 500 epochs are regarded as failure of the learning. The left shows an exhaustive investigation of a wide range of 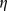 and 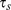, and the data are averaged over 30 runs. A small number of learning parameters are examined in the right figure, and the data are averaged over 100 runs.

We further conduct another experiment to evaluate the effects of both 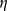 and 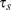 on the learning. In this experiment, a single LIF neuron with 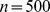 afferent neurons is considered. The neuron is trained to correctly memorize a set of 10 spike patterns randomly generated over a time window of 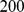 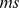. The neuron is trained in a maximum number of 500 epochs to correctly associate all these patterns with a desired spike train of [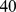, 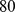, 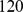, 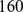] 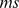. We denote that a pattern is correctly memorized if the distance between the output spike train and the desired spike train is below 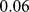. If the number of training epochs exceeds 500, we regard it as a failure. We conduct an exhaustive search over a wide range of 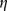 and 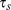. Fig. 11 shows how 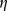 and 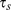 jointly affect the learning performance, which can be used as a guidance to select the learning parameters. With a fixed 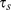, a larger 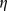 results in a faster learning speed (shown in Fig. 11, right panel), but when 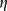 is increased above a critical value (e.g., 0.1 for 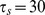 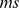 in our experiments), the learning will slow down or even fail. For small 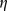, a larger 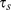 leads to a faster learning, however, for large 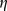, a larger 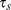 has the opposite effect. As a consequence, when 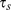 is set in a suitable range (e.g., [5], [15] 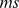), a wide range of 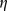 can result in a fast learning speed (e.g., below 100 epochs).

### Classification of Spatiotemporal Patterns

In this experiment, the ability of the proposed PSD rule for classifying spatiotemporal patterns is investigated by using a multi-category classification task. The setup of this experiment is similar to that in [25]. Three random spike patterns representing three categories are generated in a similar fashion to that in the previous experiments, and they are fixed as the templates. A Gaussian jitter with a standard deviation of 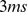 is used to generate training and testing patterns. The training set and the testing set contain 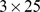 and 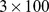 samples, respectively. Three neurons are trained to classify these three categories, with each neuron representing one category. Different neurons for each category can be specified to fire different spike trains. However, for simplicity, all the neurons in this experiment are trained to fire the same spike train (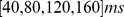). The experiment is repeated 100 times, with each run having different initial conditions.

After training, classification is performed on both the training and the testing set. In the classification task, we propose two decision-making criteria: absolute confidence and relative confidence. With the absolute confidence criterion, only if the distance between the desired spike train and the actual output spike train of the corresponding neuron is smaller than a specified value (0.5 is used here), then the input pattern will be regarded as being correctly classified. As for the relative confidence criterion, a scheme of competition is used. The incoming pattern will be labeled by the winning neuron that produces the closest spike train to its desired spike train.

**Figure 12:**
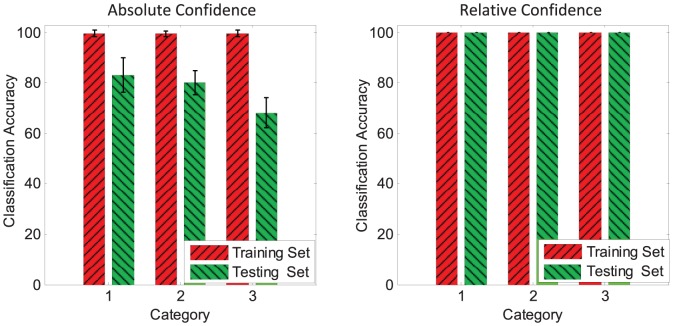
The average accuracies for the classification of spatiotemporal patterns. There are 3 categories to be classified. The average accuracies are represented by shaded bars. Two types of criteria for making decision are proposed and investigated. The left is the absolute confidence criterion, and the right is the relative confidence criterion. All the data are averaged over 100 runs.

Fig. 12 shows the average classification accuracy for each category under the two proposed decision criteria. From the absolute confidence criterion, we see that the neuron successfully classifies the training set with an average accuracy of 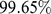. The average accuracy for the testing set is 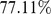. Noteworthily, under the relative confidence, both the average accuracies for the training and the testing set reach 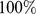. The performance for the classification task is therefore significantly improved by the relative confidence decision making criterion. With the absolute confidence criterion, the trained neuron strives to find a good match with the memorized patterns. However, with the relative confidence criterion, the trained neuron attempts to find the most likely category through competition.

For the classification of spatiotemporal patterns, the tempotron is an efficient rule [15] in training LIF neurons to distinguish two classes of patterns by firing one spike or by keeping quiescent. We use the tempotron rule to benchmark the PSD rule in the classification of spatiotemporal patterns. The tempotron rule is applied to perform the same classification task as above. The classification accuracies are shown in Table 1. As can be seen from Table 1, our proposed rule with the relative confidence criterion has a comparable performance to the tempotron rule. Moreover, the PSD rule is advantageous in that it is not limited to performing classification, but it is also able to memorize patterns by firing desired spikes at precise time.

**Table 1:** Multi-Category Classification of Spatiotemporal Patterns.

Accuracy (%)	Category 1		Category 2		Category 3	
	Training	Testing	Training	Testing	Training	Testing
Absolute Confidence	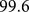	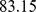	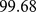	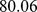	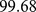	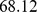
	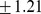	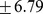	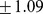	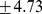	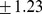	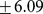
Relative Confidence	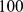	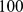	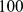	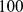	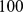	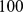
Tempotron	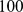	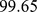	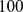	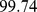	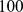	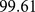
		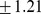		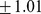		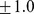

### Optical Character Recognition

**Figure 13:**
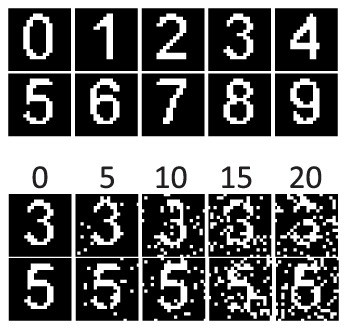
Illustration of image samples. Each image has a size of 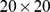 B/W pixels. The top two rows show template images. The bottom two rows show images with noise introduced to the templates. Reversal noise is used where each pixel is randomly reversed with a probability denoted as the noise level. A range of noise level of 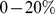 is illustrated.

In order to investigate the capability of the PSD rule over a practical problem, an OCR task is considered in this experiment. Images of digits 0-9 are used. Each image has a size of 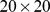 black/white (B/W) pixels. Additionally, a reversal noise is introduced to generate noisy images. We do this by reversing a pixel randomly with a probability denoted as the noise level. Fig. 13 illustrates some image samples. The digits are destroyed gradually with an increasing noise level. When the noise level is above 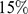, the digits are hardly recognizable.

One of the major challenges of applying SNNs to practical problems is that proper encoding methods are required to produce the input data [26], [41]. Encoding is the first step of utilizing spiking neurons. It aims to generate spike patterns that represent the external stimuli. However, how the external information is encoded in the brain still remains unclear. Many encoding mechanisms have been proposed for converting images into spikes such as rate code [21], latency code [26], [42] and phase code [27], [43]. The rate code is unsuitable for the rules that learn precise spike patterns. A direct utilization of the latency code is also found to be inappropriate. For example, if a simple latency code is used in this OCR task, the spikes in the input spatiotemporal pattern will all occur at 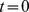 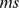. This does not work for spatiotemporal mapping algorithms including PSD, ReSuMe [23], Chronotron [24] and SPAN [25]. These spatiotemporal mapping algorithms cannot guarantee successful learning of an arbitrary spatiotemporal spike pattern. To trigger a desired spike, a sufficient number of input spikes around it are required. Long delays will not be effectively learnt since the causal connection could not be built. In real nervous systems, neurons rarely fire in such a highly synchronized manner but rather in a distributed one [7], [8], [44]. Thus, proper encoding is required not only for successful learning association but also for maintaining some level of biological realism.

**Figure 14:**
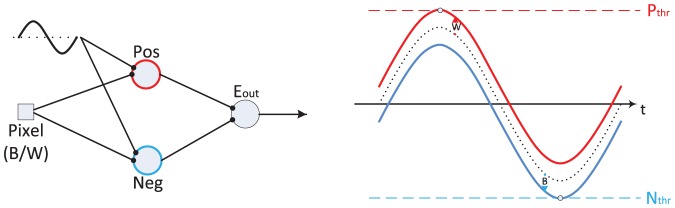
Illustration of the encoding schema. The left shows the structure of an encoding unit. The encoding unit includes a positive neuron (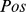), a negative neuron (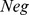) and an output neuron (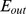). Each encoding unit is assigned to a subthreshold membrane oscillation. Both 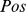 and 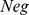 neurons receive signals from this subthreshold membrane oscillation and the corresponding pixel. The 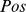 neuron only reacts to positive activation voltage, while the 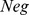 neuron only reacts to negative activation voltage. The firing of either the 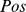 neuron or the 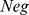 neuron will immediately cause the firing of the 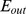 neuron. The right illustrate the dynamics of the encoding. The B/W pixel will cause a downward/upward shift from the subthreshold membrane oscillation. A spike is generated if the membrane potential crosses the threshold line (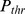 and 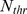).

An increasing body of evidence shows that action potentials are related to the phases of the intrinsic subthreshold membrane potential oscillations [45]–[47]. These observations support the hypothesis of a phase code. Following the phase code presented in [27], [43], we develop a simple encoding method for this task. The mechanism of our encoding model is illustrated in Fig. 14. The encoding unit consists of a positive neuron (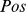), a negative neuron (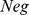) and an output neuron (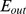). Each encoding unit is connected to a pixel and a subthreshold membrane potential oscillation. For simplicity, the oscillation for the 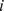-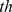 encoding unit is described as:
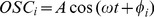
(13)where 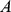 is the magnitude of the subthreshold membrane oscillation, 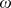 is the phase angular velocity and 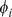 is the initial phase. 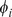 is defined as:
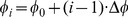
(14)where 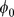 is the reference phase and 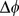 is the phase difference between nearby encoding units. We set 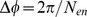 where 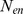 is the number of encoding units. 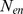 is equal to the number of pixels in the image (400 here). The oscillation period is set to be 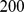 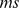 which corresponds to a frequency of 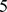 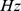.

The 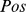 neuron only responds to positive activation potential, while the 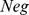 neuron only reacts to negative activation potential. An input of B/W pixel will cause a downward/upward shift from the subthreshold membrane oscillation. Whenever the membrane potential crosses the threshold, a spike is generated. Through the fine tuning of parameter 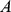, the amount of shift and the threshold values, we set the spike to occur at peaks of the oscillation. The firing of either the 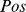 neuron or the 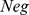 neuron will immediately trigger the firing of the 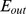 neuron. The encoding units therefore output a spike at one phase for a white pixel and another shifted phase of 180 degrees for a black pixel. Also, the emitted phases change depending which pixel is the input.

We select 10 neurons to learn the patterns generated by the encoding units. Each learning neuron corresponds to one category. The parameter setting of the learning neurons is the same as that in the previous task of spatiotemporal pattern classification. Each neuron is trained to produce a target spike train (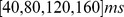) when a pattern from the assigned class is presented, and not to spike when patterns from other classes are presented. In principle, different target spike trains can be used for different digits. The neurons are trained for 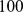 epochs. In each training epoch, a training data set of 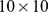 samples is formed. There are 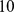 samples for each digit. Among these 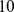 samples, one is the template image and the other 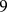 are generated with a random noise level of 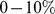. After training, the neurons are tested on different noise levels. On each noise level, 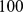 noise patterns are generated for each digit. The relative confidence criterion is used for making decision. In our test, the category of an input pattern will be decided by one of the neurons that generates the lowest spike distance.

**Figure 15:**
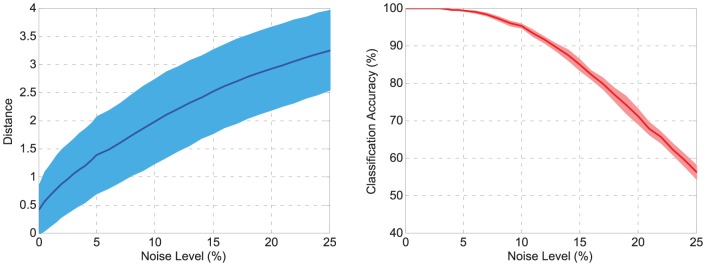
Performance on OCR task. The left shows the association ability of the neuron to map a typical digit with the desired spike train. Digit “8” is used as an example here. The distance between the output spike train and the desired spike train is depicted versus the noise level. The right shows the classification accuracy on the testing set. Solid lines denote the average and shaded areas denote the standard deviation. All the data are averaged over 30 runs.

Fig. 15 shows the testing results. In order to observe the association ability of the neuron to map a digit with the desired spike train, digit “8” is used as an example. The neuron corresponding to digit “8” can successfully produce a spike train close to the target train when the noise level is low. This association worsens as the noise level increases. As shown in Fig. 15, the classification accuracy remains high when the noise level is low and will drop gradually with increasing noise level. Even when the image is seriously damaged by the noise (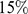 noise level), a high accuracy of around 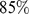 can still be obtained. The results show that the trained neurons can successfully associate the template images with the target spike train. Moreover, the trained neurons present a high recognition ability under the relative confidence criterion even if images are damaged by noise.

## Discussion

The PSD rule is proposed for the association and recognition of spatiotemporal spike patterns. In summary, the PSD rule transforms the input spike trains into analog signals by convolving the spikes with a kernel function. By using a kernel function, the analog signals are presented in the simple form of synaptic currents. It is biologically plausible because it allows us to interpret the signals with physical meaning. Synaptic adaptation is driven by the error between the desired and the actual output spikes, with positive errors causing LTP and negative errors causing LTD. The amount of synaptic adaptation is determined by the transformed signal of the input spikes (postsynaptic currents here) at the time of modification occurrence. When the actual spike train is the same as the desired spike train, the adaptation of the weights will be terminated.

There is a supervisory signal involved in the PSD rule. The most documented evidence for supervised rules comes from studies of the cerebellum and the cerebellar cortex [18], [19]. It is shown that supervisory signals are provided to the learning modules by sensory feedback [20] or other supervisory neural structures in the brain [19]. A neuromodulator released by the supervisory system can induce the control of the adaptation. This control occurs for several neuromodulatory pathways, such as dopamine and acetylcholine [48], [49]. Experimental evidence shows that N-methyl-D-aspartate (NMDA) receptors are critically involved in the processes of LTP and LTD [50]-[52]. After opening the NMDA channels, the resulting 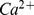 entry then activates the biochemistry of potentiation which leads to LTP [52]. Suppression of NMDA receptors by spike-mediated calcium entry may be a necessary step in the induction of LTD [52], [53]. The synaptic modification can be implemented through a supervisory control of opening or suppression of these NMDA channels.

The PSD rule is simple and efficient in synaptic adaptation. Utilizing the postsynaptic current as the eligibility trace for weight adaptation is a simple and efficient choice. The same signals of postsynaptic currents are also used in the synaptic adaptation as in the neuron dynamics, unlike the learning rules such as [22], [25], [28] where different sources of signals were used. Thus, the number of signal sources involved in the learning is reduced, which will directly benefit the computation. Secondly, unlike the arithmetic-based rules [22], [24], [25], where a complex error calculation is required for the synaptic adaptation, the PSD rule is based on a simple form of spike error between the actual and the desired spikes. The synaptic adaptation is driven by these precise spikes without complex error calculation. As a matter of fact, the weight modification only depends on currently available information (shown as Fig. 2). Additionally, due to the ability of the PSD rule to operate online, it is suitable for real-time applications. According to the PSD rule, different kernels, such as the exponential kernel and 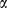 kernel, can also be used in convolving the spikes to provide different eligibility traces.

The PSD rule is designed for processing spatiotemporal patterns, where the exact time of each spike is used for information transmission. The PSD rule is unsuitable for learning patterns under the rate code because this rule is designed to process precise-timing spikes by its nature. The rate code uses the spike count but not the precise time to convey information. Like other spatiotemporal mapping algorithms, including ReSuMe [23], Chronotron [24] and SPAN [25], the PSD rule cannot guarantee successful learning of an arbitrary spatiotemporal spike pattern. A sufficient number of input spikes around the desired time are required for establishing causal connections. In other words, the temporal range covered by the desired spikes should be covered by the input spikes.

The spiking neurons are equivalent to traditional neurons such as perceptron under certain conditions [15], [54]. The running of a spiking neuron is a continuous process over a period of time while a perceptron does not involve the concept of time. However, the common feature between perceptrons and spiking neurons is that the calculation of a weighted sum is needed. Segmenting the running time of the spiking neuron into several fixed points, the perceptron can replace the spiking neuron. The input vectors for the perceptron are the postsynaptic currents at these fixed time points. According to [54], the supervised association can be transformed into a classification problem and then be solved by using the perceptron learning rule. The target of the classification is to distinguish the spike firing time from non-spike firing time. However, a large number of fixed points are required for the perceptron to achieve similar dynamics of the spiking neuron. This means the perceptron needs to remember all pattern vectors at these fixed points. The computational power of spiking neurons is sacrificed by using perceptrons at this point.

In all the experiments, a single spike code is used for afferent neurons, where each input neuron only fires a single spike during the entire time window. This single spike code is chosen for various reasons but more than one spike is also allowed for the PSD rule. Firstly, a single spike code is simple for analysis and efficient for computation [22], [26]. Secondly, there is strong biological evidence supporting the single spike code. Visual systems can analyze a new complex scene in less than 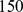 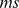 [44], [55]. This period of time is impressive for processing, considering the billions of neurons involved. This suggests neurons exchange only one or few spikes. Single spike codes can also fit situations where information is coded in the time of the first spike, relative to the onset of stimuli [56], or situations where information is coded relative to a background oscillation [24], [47]. The PSD rule is also suitable for multi-spike train (results shown in Fig. 6). When the number of spikes from each afferent neuron is not high enough, the neuron can produce the desired spike train after several epochs. When the number of spikes increases, the learning becomes slower and more difficult to converge. Additionally, the biological plausibility of an encoding scheme that can use multiple spikes to code information is still unclear.

### Related Works

Several learning algorithms have been proposed to explore how spiking neurons may respond for processing and memorizing spatiotemporal patterns.

The tempotron rule [15] is one such learning rule where neurons are trained to discriminate between two classes of spatiotemporal patterns. This learning rule is based on a gradient descent approach. In the tempotron rule, the synaptic plasticity is governed by the temporal contiguity of presynaptic spike and postsynaptic depolarization and a supervisory signal. The neurons could be trained to successfully distinguish two classes by firing a spike or by remaining quiescent. However, the neurons do not learn to fire at precise time. Since the tempotron rule mainly aims at decision-making tasks, it cannot support the same coding scheme used in both the input and output spikes. To support the same coding scheme through the input and output, a learning rule is needed to let the neuron not only fire but also fire at the specified time. In addition, the tempotron is designed for a specific neuron model, which might limit its usage on other spiking neuron models. For the decision-making task (classification), our proposed rule can obtain a comparable performance as the tempotron rule (see Table 1).

SpikeProp [22] is a supervised learning rule for SNNs that can solve nonlinear classification problems by emitting a single spike at the desired time. The major limitation is that SpikeProp and its extension in [57] do not allow multiple spikes in the output spike train. Thus, several different learning rules have been developed to train neurons to produce multiple output spikes in response to a spatiotemporal stimulus, such as ReSuMe [23], [28], Chronotron [24] and SPAN [25], as well as our PSD rule.

In both the SPAN rule and the Chronotron E-learning rule, the synaptic weights are modified according to a gradient descent approach in an error landscape. The error function in the Chronotron is based on the Victor 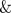 Purpura (VP) distance [58], while in the SPAN rule the error function is based on a metric similar to the van Rossum metric [38]. These arithmetic calculations can easily reveal why and how networks with spiking neurons can be trained, but the arithmetic-based rules are not a good choice for designing networks with biological plausibility. The biological plausibility of error calculation is at least questionable. In contrast, the PSD minimizes the error between the actual output spike train and the desired spike train without the need for an explicit gradient calculation. Without extra calculation of the error, the PSD provides an efficient way for processing spatiotemporal patterns. On the other hand, since the PSD rule is derived from the common WH rule, it can also easily reveal why and how neurons can be trained similarly with arithmetic-based rules.

From the perspective of increased biological plausibility, the Chronotron I-learning rule and the ReSuMe rule are considered below. The I-learning rule was heuristically defined in [24] where synaptic changes depend on the synaptic currents. This learning rule is quite similar to the PSD rule and it can be considered as a variation of the PSD rule. According to the I-learning rule, its development seems to be based on a particular case of the Spike Response Model [1], which might also limit its usage on other spiking neuron models or at least is not clearly demonstrated. Moreover, those synapses with zero initial weights will never be updated according to the I-learning rule. This will inevitably lead to information loss from those afferent neurons. In the PSD rule, all these issues are considered. The PSD is a more general rule and it is analytically derived. Through careful choice, the eligibility trace in the PSD rule can be represented by the postsynaptic current. In the tempotron rule, the postsynaptic voltage is involved in the learning. We refer to both the postsynaptic current and the postsynaptic voltage as the postsynaptic state. A crucial role of the postsynaptic state in the induction of long term plasticity has been demonstrated in [50]–[52]. Similar to the PSD rule and the SPAN rule, the ReSuMe rule is derived from the WH rule. The ReSuMe interprets the WH rule as a combination of a Hebbian and an anti-Hebbian process within a learning window. It was demonstrated in [25] that the form of the SPAN rule has a surprising similarity to the ReSuMe rule with an exponential kernel. Similarly, we can transform the PSD rule by replacing the kernel used in Eq. (11) with the exponential kernel. This leads to:
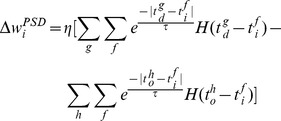
(15)


A batch learning version of the ReSuMe rule given in [24] is described as:
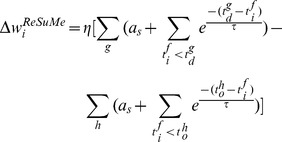
(16)where 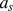 is a non-Hebbian term used for speeding up the convergence of the learning.

As can be seen from the above equations, the PSD rule is also mathematically similar to the ReSuMe rule under certain conditions. The similarity among PSD, SPAN and ReSuMe results from the common WH rule. All these rules are derived from the WH rule with different interpretations.

Surprisingly, the WH rule also guarantees an intrinsical similarity among other learning rules such as synaptic scaling rules [59], [60]. For example, a synaptic scaling rule was introduced in [60] as:
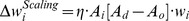
(17)where the variable 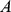 measures the average activity of neurons, and it can be referred to as the firing rate. If a kernel with a long time constant is used to convolve the input, the actual output and the desired spikes, a similar measurement of the average firing activity will be obtained. Thus, the common WH rule can be presented in a similar form as the scaling rule.
